# Destruction of vascular endothelial glycocalyx during formation of pre-metastatic niches

**DOI:** 10.1016/j.heliyon.2024.e29101

**Published:** 2024-04-01

**Authors:** Rui Qu, Wenxuan Du, Shuyao Li, Wei Li, Guangfei Wei, Zhoujiang Chen, Huile Gao, Sanjun Shi, Liang Zou, Hanmei Li

**Affiliations:** a School of Food and Biological Engineering, Chengdu University, Chengdu, 610106, China; b Sichuan Industrial Institute of Antibiotics, School of Pharmacy, Chengdu University, Chengdu, 610106, China; c School of Pharmacy, Chengdu University of Traditional Chinese Medicine, Chengdu, 611137, China; d Key Laboratory of Drug-Targeting and Drug Delivery System of the Education Ministry, Sichuan Engineering Laboratory for Plant-Sourced Drug and Sichuan Research, Center for Drug Precision Industrial Technology, West China School of Pharmacy, Sichuan University, Chengdu 610041, China; e Clinical Medical Research Center, Zhenjiang Hospital of Integrated Traditional Chinese and Western Medicine, Zhenjiang, 212004, China

## Abstract

A special microenvironment called the “pre-metastatic niche” is thought to help primary tumor cells migrate to new tissues and invade them, in part because the normal barrier function of the vascular endothelium is compromised. While the primary tumor itself can promote the creation of such niches by secreting pro-metastatic factors, the underlying molecular mechanisms are still poorly understood. Here, we show that the injection of primary tumor-secreted pro-metastatic factors from B16F10 melanoma or 4T1 breast cancer cells into healthy mice can induce the destruction of the vascular endothelial glycocalyx, which is a polysaccharide coating on the vascular endothelial lumen that normally inhibits tumor cell passage into and out of the circulation. However, when human umbilical vein endothelial cultures were treated *in vitro* with these secreted pro-metastatic factors, no significant destruction of the glycocalyx was observed, implying that this destruction requires a complex *in vivo* microenvironment. The tissue section analysis revealed that secreted pro-metastatic factors could clearly upregulate macrophage-related molecules such as CD11b and tumor necrosis factor-α (TNF-α) in the heart, liver, spleen, lung, and kidney, which is associated with the upregulation and activation of heparanase. In addition, macrophage depletion significantly attenuated the degradation of the vascular endothelial glycocalyx induced by secreted pro-metastatic factors. This indicates that the secreted pro-metastatic factors that destroy the vascular endothelial glycocalyx rely primarily on macrophages. Our findings suggest that the formation of pre-metastatic niches involves degradation of the vascular endothelial glycocalyx, which may hence be a useful target for developing therapies to inhibit cancer metastasis.

## 1 Introduction

Tumor metastasis can substantially increase the risk of poor prognosis, including mortality, and it can be difficult to treat with surgery, radiotherapy, or chemotherapy [1,2]. Malignant tumors secrete numerous factors that can create “pre-metastatic niches” in secondary tissues to recruit tumor cells and promote their colonization at new locations [[3], [4], [5]]. The pre-metastatic niche (PMN) is a special microenvironment associated with inflammation, and its formation is often accompanied by inflammation [[6], [7], [8]]. Pre-metastatic niches contain abundant inflammatory factors, the vascular endothelial cells express high levels of adhesion molecules to which tumor cells can bind, and the vascular endothelium exhibits “leakiness” that facilitates the intra- and extravasation of tumor cells, allowing them to travel to and invade new tissues [[9], [10], [11]]. Pre-metastatic niches thus act as the “soil” for circulating tumor cells, which serve as the “seed” that will grow into clinically significant metastasis [[12], [13], [14]].

Vascular endothelial glycocalyx (VEG) is a polysaccharide composite located on the surface of vascular endothelial cells [15]. VEG covers the surface of endothelial cells, constitutes an electrical and mechanical barrier that restricts the passage of plasma proteins, regulates the fluid balance between blood vessels and tissue space, and prevents blood cells from adhering to the vascular endothelium [16,17]. Therefore, the VEG is a selective permeability barrier of blood vessel walls and is especially important for maintaining the normal microenvironment of the vascular endothelium. Vascular endothelial glycocalyx destruction has been implicated in various diseases [[18], [19], [20]]. For example, in acute lung injury, its destruction permeabilizes the lung vasculature, leading to pulmonary edema and neutrophil transendothelial migration, which extravasate into the surrounding tissue and release numerous pro-inflammatory factors [[21], [22], [23]].

**Fig. 1:**
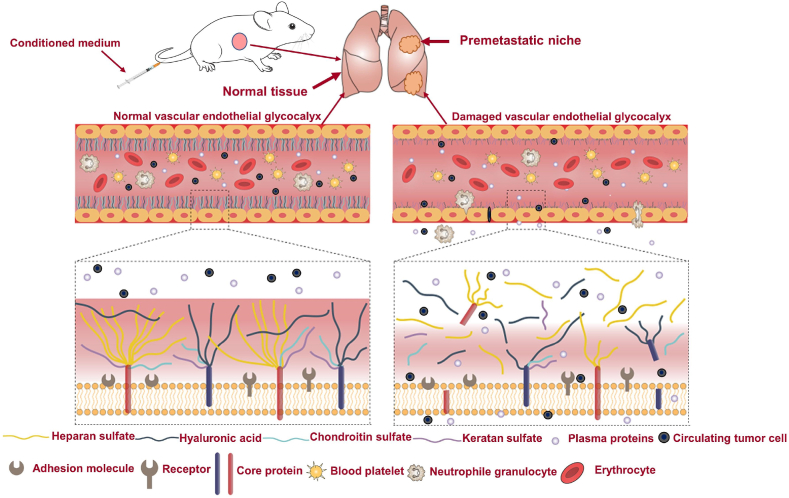
Illustration of our hypothesis that the vascular endothelial glycocalyx (VEG) degrades during pre-metastatic niche formation.

During metastasis, cancer cells migrate out of blood vessels and require initial homing, adhesion, and crossing of the endothelium, which occurs due to dysfunction of the vascular endothelium. The destruction of the vascular endothelial glycocalyx may be an important step in the formation of a pre-metastatic niche. Normally, the vascular endothelial glycocalyx shields various adhesion molecules that are highly expressed on the vascular endothelium, thereby preventing blood cells and circulating tumor cells from adhering to the vessel walls and gaining access to the surrounding tissue. During pre-metastatic niche formation, destruction of the vascular endothelial glycocalyx can facilitate the intra- and extravasation of primary tumor cells en route to secondary tissues (Fig. 1).

We explored this hypothesis by injecting conditioned medium from cancer cell line cultures into mice and then assaying the plasma levels of hyaluronic acid (HA), syndecan-1 (SD), and heparan sulfate (HS), which are components of the vascular endothelial glycocalyx and, therefore, markers of its integrity. Conditioned medium containing abundant tumor cell-derived cytokines and exosomes can induce a pre-metastatic microenvironment that facilitates circulating tumor cell seeding and metastasis [24,25]. We also investigated the mechanism underlying the destruction of vascular endothelial glycocalyx [26,27]. Our results suggest that a conditioned medium can induce vascular endothelial glycocalyx destruction during the formation of pre-metastatic niches and that repairing the vascular endothelial glycocalyx may be a therapeutic approach against cancer metastasis.

## 2 Materials and methods

### 2.1 Materials, reagents, cells, and animals

Dulbecco's modified Eagle's medium (DMEM) and RPMI 1640 were purchased from Thermo Fisher Scientific (Waltham, MA, USA). Fetal bovine serum was purchased from Shanghai Titan Technology (Shanghai, China). Penicillin-streptomycin liquid and horseradish peroxidase-conjugated goat anti-rabbit IgG (H + L) were purchased from Solabor Biotechnology (Beijing, China). Green fluorescence-labeled wheat germ agglutinin (FITC-WGA) was purchased from Xi'an Ruixi Biotechnology (Xi'an, China). 3-(4,5-dimethyl-2-thiazole)-2,5-diphenyl-2H-tetrazolethiazolium bromide (MTT) was purchased from Beijing Bailinway Technology (Beijing, China). Glutaraldehyde (50% v/v) and lanthanum nitrate were purchased from Maclin Biochemical Technology (Shanghai, China). Sodium cacodylate was purchased from Shanghai Yien Chemical Technology (Shanghai, China). An enzyme-linked immunosorbent assay (ELISA) kit to measure hyaluronic acid in mice was purchased from Jiangsu Jing Mei Biotechnology (Jiangsu, China), ELISA kits to measure heparan sulfate and syndecan-1 were purchased from Boyan Biotechnology (Shanghai, China). Rabbit anti-Heparanase-1, anti-TNF-alpha, and anti-CD11b antibodies were purchased from Bioss antibodies (Beijing, China). Clodronate liposomes were purchased from Yeasen Biotechnology (Shanghai, China).

The mouse breast cancer cell line 4T1, mouse melanoma cell line B16F10, and human umbilical vein endothelial cells (HUVECs) were obtained from the Shanghai Institute of Biological Sciences (Shanghai, China).

Male BALB/c and C57 mice (3–4 weeks old) weighing approximately 20 g were purchased from Spaifu Biotechnology (Beijing, China). All animal experiments were performed in accordance with the principles of care and use of laboratory animals and approved by the Experimental Animal Ethics Committee of Chengdu University.

### 2.2 Cell culture and harvesting of conditioned medium

HUVECs were cultured in DMEM supplemented with 10% (v/v) fetal bovine serum, 100 mg/mL streptomycin, and 100 U/mL penicillin. 4T1 and B16F10 cells were cultured in RPMI 1640 medium in the same manner. The cultures were maintained in a humidified incubator at 37 °C in an atmosphere containing 5% CO_2_. When the cultures reached 80%–90% confluence, they were treated with 0.25% trypsin containing 0.01% ethylenediaminetetraacetic acid, harvested, centrifuged, resuspended in fresh medium containing 10% fetal bovine serum, and split 1:2 into new culture vessels.

After the cultures reached confluence, the medium was replaced with RPMI 1640 without fetal bovine serum, and the cells were cultured overnight. The conditioned medium was harvested and centrifuged at 1000×*g* at 4 °C for 3 min, and the supernatant was passed through a 0.22 μm filter and stored at −20 °C until further use [28]. In order to ensure the same concentration of conditioned medium in each experiment, a large amount of conditioned medium was collected and stored at −20 °C until further use.

### 2.3 Mouse models of pre-metastatic niches

Clarified conditioned medium (200 μL) from 4T1 cells or B16F10 cells, obtained as described in Section 2.2, was injected into BALB/c or C57 mice, respectively, via the tail vein in order to induce pre-metastatic niches The animals were provided ad libitum access to normal food and water before and after injection of the conditioned medium.

### 2.4 Analysis of vascular endothelial glycocalyx components in mouse plasma

At 12 h before injection of conditioned medium, blood (100 μL) was collected from the eye socket of mice into a microcentrifuge tube containing 10 μL of 0.5% heparin in physiological saline. The plasma was centrifuged at 2000×*g* at 4 °C for 20 min, and the supernatant was assayed for heparan sulfate, hyaluronic acid, and syndecan-1 using commercial ELISA kits and enzyme labels [29,30]. The blood sampling and assays were repeated at 4, 8, and 24 h after injection of the conditioned medium.

### 2.5 Histopathology of the major organs

At 24 h after injection with the conditioned medium, the animals were euthanized and the major organs were collected, washed with 0.9% physiological saline, and then fixed for 24 h with 4% (v/v) paraformaldehyde. The tissues were dehydrated with a graded series of alcohol solutions that also contained benzene and xylene, embedded in wax, sectioned to a thickness of 3 μm, baked at 60 °C, dewaxed, washed in pure water, washed with water after immersion in a graded series of alcohol solutions containing xylene, stained with hematoxylin for 4 min, differentiated with 1% (v/v) hydrochloric acid in 75% (v/v) alcohol, followed by 1% (v/v) ammonia solution to revert it to blue, washed again with pure water, stained with eosin for 2 min, washed in pure water, dehydrated, allowed to air-dry, and sealed with neutral gum [31,32]. The sections were observed under a microscope.

### 2.6 Tumor lung metastasis after injection of conditioned medium

Conditioned medium from 4T1 cells or PBS was injected as 200 μL (4 mL/kg) once a day into the tail veins of BALB/c mice. Three days later, the mice were injected with a 4T1 cell suspension (200 μL containing 1 × 10^6^ cells/mL). At 18 days after the tumor inoculation, the mice were killed and their lungs were removed, stained with Bouin's solution (saturated picric acid solution (1.22%) 75 mL, glacial acetic acid 5 mL, and formaldehyde 25 mL) at 37 °C for 6 h, rinsed with 75% (v/v) ethanol, photographed, and the nodules counted.

### 2.7 Expression of heparanase, TNF-α, and CD11b in the major organs

Paraformaldehyde-fixed, paraffin-embedded tissue sections were prepared as described in Section 2.5, subjected to antigen recovery by incubation in citric acid buffer (pH 6.0), boiled in a microwave oven, allowed to cool for 8 min, and then heated slightly for 7 min. The sections were allowed to cool to room temperature and were washed three times (5 min each) in phosphate-buffered saline (PBS) on a shaker. The sections were incubated in 3% hydrogen peroxide at room temperature for 25 min in the dark and then washed three times (5 min each) in PBS on a shaker. The sections were incubated at room temperature for 30 min in 5% normal goat serum in PBS, overnight at 4 °C with primary antibody (rabbit anti-Heparanase-1 antibody, rabbit anti-TNF-alpha antibody, rabbit anti-CD11b antibody), washed three times in PBS on a shaker as above, and then incubated at room temperature for 50 min with secondary antibody (HRP conjugated goat anti-rabbit IgG (H + L)) conjugated to horseradish peroxidase. Finally, the sections were washed three times in PBS on a shaker and developed in DAB solution. The sections were counterstained with hematoxylin (30 s), 1% hydrochloric acid (a few seconds), and ammonia water, as described in Section 2.5 [33].

### 2.8 Assessment of vascular endothelial glycocalyx in the major organs based on FITC-WGA staining

At 24 h after injection of conditioned medium, the major organs were harvested from animals, washed with 0.9% physiological saline, coated with a layer of OCT embedding gel, and placed at 4 °C for 5–10 min to allow the OCT gel to soak into the tissue. The sections were then frozen on a quick-freezing rack; another layer of OCT was overlaid and allowed to solidify; and thin sections were prepared, stained for 40 min with 20 μg/mL FITC-WGA, rinsed with PBS, and visualized under a fluorescence microscope.

### 2.9 Assessment of vascular endothelial glycocalyx in the major organs based on transmission electron microscopy

As the lung is the major site of metastasis for many types of cancer [[34], [35], [36]], we excised lungs from mice at 24 h after injection of conditioned medium, after which they were washed with 0.9% physiological saline, sectioned, and fixed for at least 2 h at 4 °C with electron microscopy fixative containing 2% lanthanum nitrate (47.50 mL 0.2 mol/L sodium cacodylate, 3.99 mL 0.2 mol/L HCl, purified water 138.51 mL, 10 mL 50% (v/v) glutaraldehyde, and 5.33 g lanthanum nitrate) to label the vascular endothelial glycocalyx. The samples were rinsed three times (15 min each) with 0.1 M phosphoric acid buffer (pH 7.0), fixed with 1% osmic acid solution for 2–4 h, rinsed again three times with 0.1 M phosphoric acid buffer, dehydrated with a graded ethanol series, treated with 100% ethanol for 20 min, and finally transferred to pure acetone for 20 min. The samples were treated for 1 h with a mixture of the embedding agent and acetone (1:1, v/v), and then with a 3:1 mixture for 3 h, followed by treatment with the pure embedding agent for 12 h. The samples were heated to 70 °C for 8–12 h, sectioned to a thickness of 70–90 nm, and then stained for 5 min with lead citrate solution and a further 5 min with a saturated solution of 50% uranium-oxyacetate acetaldehyde. The samples were then dried and analyzed using a transmission electron microscope [[37], [38], [39]].

### 2.10 Effects of conditioned medium on cultured HUVECs

HUVECs were seeded in 96-well plates (10^4^ cells/well), cultured for 24 h, washed twice with PBS, and cultured for 12 h in fresh medium containing various concentrations of conditioned medium. Cell viability was determined by MTT assay, whereby the absorbance at 490 nm was measured using a multifunctional enzyme labeling instrument. Alternatively, the treated cells were stained for 1 h with FITC-WGA (2 μg/mL, 100 μL) to label vascular endothelial glycocalyx, washed twice with PBS, fixed with 4% paraformaldehyde for 15 min, washed twice with PBS, and analyzed under a fluorescence microscope. As a positive control, the cells were stimulated with lipopolysaccharide, which is known to degrade the vascular endothelial glycocalyx [40].

We conducted this experiment to directly observe whether conditioned media could cause vascular endothelial glycocalyx shedding at the cellular level. HUVECs were inoculated in 24-well plates with 500 μL containing 1 × 10^5^ cell/mL cell suspension per well. After 24 h, the culture medium was removed and the cells were washed twice with PBS. Blood-free culture medium and 100 μL of 1 μg/mL LPS was added, and 100 μL conditioned medium diluents at various concentrations were used. The cells were then incubated for 12 h. The culture medium was removed, and the cells were washed twice with PBS. This was followed by the addition of 100 μL of 2 μg/mL FITC-WGA, and the cells were then incubated for 1 h to label the vascular endothelial glycocalyx with FITC-WGA. The cells were washed twice with phosphate-buffered saline (PBS), fixed with 4% paraformaldehyde for 15 min, and then washed twice with PBS. The vascular endothelial glycocalyx status in each group was observed using a fluorescence microscope.

### 2.11 Analysis of vascular endothelial glycocalyx components in mouse plasma after macrophage clearance

Male BALB/c mice were intravenously injected with 200 μL (4 mL/kg) of macrophage scavenger (clodronate liposomes, CL) followed by injection with the same dose of clodronate liposomes 24 h later, and conditioned medium from 4T1 cells was then injected intravenously 4 h after the second injection. At 24 h after injection of the 4T1 cell conditioned medium, orbital blood was collected. The vascular endothelial glycocalyx components were detected using the methods described in Section 2.4.

### 2.12 Statistical analysis

The data are reported as means ± the standard deviation unless indicated otherwise. Intergroup differences were assessed for significance using Student's *t-*test and were considered significant if *P* < 0.05.

## 3 Results

### 3.1 Conditioned medium from cancer cell cultures can induce the formation of pre-metastatic niches

**Fig. 2:**
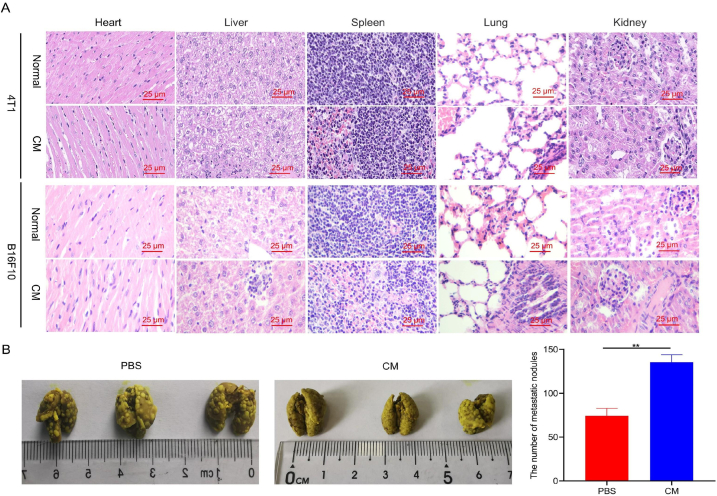
Conditioned medium induced the formation of pre-metastatic niches. A. Hematoxylin and eosin (H&E) staining of the major organs from healthy mice after injection with conditioned medium from 4T1 or B16F10 cell cultures. Scale bar, 25 μm. B. Representative images of lungs with metastatic nodules stained with Bouin's solution, and statistics on the number of nodules are presented. **p < 0.01.

According to a previous report, the pre-metastatic niche is a specialized microenvironment with aberrant changes related to inflammation [41] that allows colonization by circulating tumor cells (CTCs). Here, we studied the pathological changes in the main organs after the injection of conditioned medium to evaluate the formation of pre-metastatic niches. As shown in Fig. 2A, the injection of conditioned medium into healthy mice induced clear inflammation in the major organs, which was most severe in the lungs. Degeneration and necrosis of alveolar epithelial or interstitial cells were observed in the lung tissue. The observed inflammation was consistent with the induction of pre-metastatic niches.

In addition, we evaluated conditioned medium-induced pre-metastatic niche formation in a lung metastasis model. As shown in Fig. 2B, the yellowish-white nodules in the conditioned medium group covered almost the entire lung surface, and their numbers were much higher than those in the PBS group. These results confirm that the conditioned medium stimulated the establishment of pre-metastatic niches and promoted tumor metastasis.

### 3.2 The formation of pro-metastatic niches in mice accompanied by vascular endothelial glycocalyx degrading

**Fig. 3:**
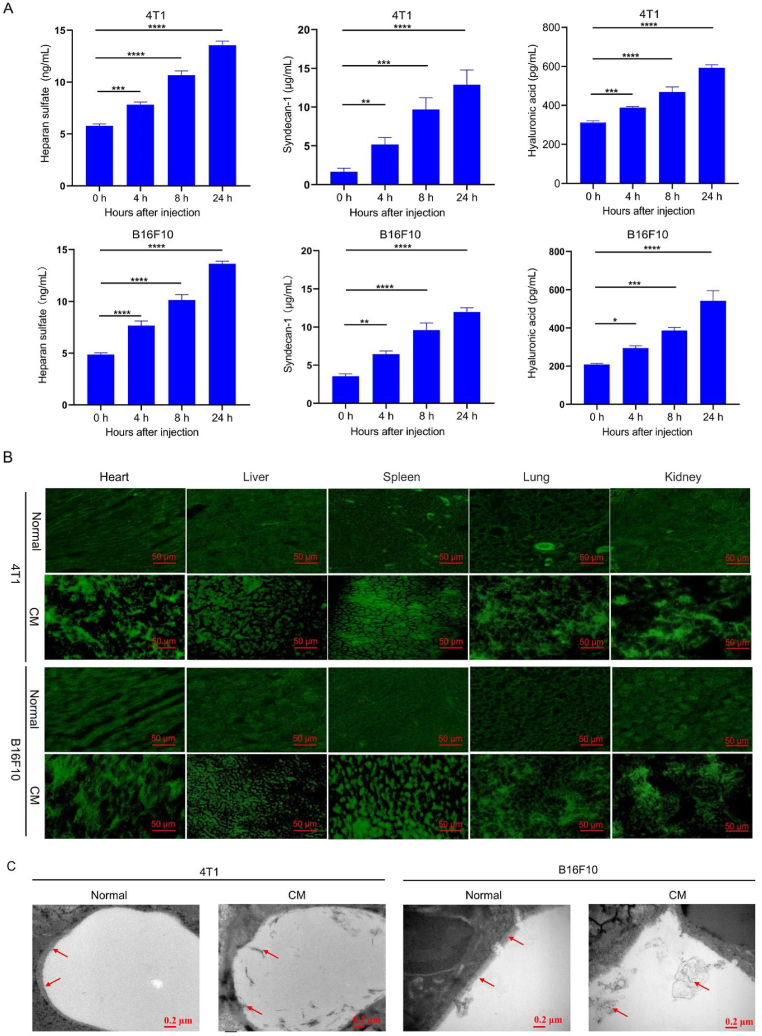
Degradation of vascular endothelial glycocalyx in mice after injection with cancer cell-secreted pro-metastatic factors. A. Concentrations of vascular endothelial glycocalyx degradation markers in the blood of healthy mice after injection of conditioned medium from 4T1 and B16F10 cell cultures at various time points. *p < 0.05, **p < 0.01, ***p < 0.001, ****p < 0.0001. B. Integrity of the vascular endothelial glycocalyx associated with the indicated organs in healthy mice after injection of conditioned medium from 4T1 or B16F10 cell cultures. The tissue sections were stained with fluorescein isothiocyanate-labeled wheat germ agglutinin or with phosphate-buffered saline (PBS). Scale bar, 50 μm. C. Representative transmission electron micrographs of the lungs from healthy mice after injection with conditioned medium from 4T1 or B16F10 cell cultures. Red arrows indicate vascular endothelial glycocalyx. Scale bar, 0.2 μm.

The vascular endothelial glycocalyx comprises proteoglycans, such as syndecans, glycoproteins, and glycosaminoglycans, which anchor soluble components to the surface of endothelial cells. Glycosaminoglycans are composed of heparan sulfate, chondroitin sulfate, and hyaluronic acid [15,42,43]. Vascular endothelial glycocalyx degradation is often assayed by measuring the levels of heparan sulfate, hyaluronic acid, and syndecan-1 in the blood [[44], [45], [46]]. As shown in Fig. 3A, the levels of all three vascular endothelial glycocalyx components increased gradually in the two types of mouse blood after the injection of the conditioned medium containing pro-metastatic factors, indicating degradation of the vascular endothelial glycocalyx.

At 24 h after the injection of the conditioned medium, the organs (heart, liver, spleen, lung, and kidney) of the mice were harvested to assess the integrity of the vascular endothelial glycocalyx. The vascular endothelial glycocalyx in tissue sections was stained with FITC-labeled wheat germ agglutinin. Wheat germ agglutinin binds specifically to glycoproteins or glycolipids containing sialic acid and *N*-acetylglucosamine residues, and fluorescently labeled agglutinin is useful for labeling the vascular endothelial glycocalyx [[47], [48], [49]]. As shown in Fig. 3B, the vascular endothelial glycocalyx in the normal group was evenly distributed in the various tissues, but it exhibited obvious aggregation, shedding, and uneven distribution in the groups injected with conditioned medium. These results indicate that the injection of the conditioned medium containing pro-metastatic factors can significantly reduce the shedding of the vascular endothelial glycocalyx associated with all major organs examined, consistent with vascular endothelial glycocalyx degradation.

Malignant tumors can affect various organs via hematogenous metastasis, with the lungs being the most affected. Here, we directly observed damage to the vascular endothelial glycocalyx in the lung by transmission electron microscopy after injection of the conditioned medium containing pro-metastatic factors for 24 h. Prior to observation, lung sections were stained with lanthanum nitrate, which binds to the vascular endothelial glycocalyx [50,51]. As shown in Fig. 3C, while the vascular endothelial glycocalyx in the lungs from the normal group appeared as villous structures in the vascular lumen, the vascular endothelial glycocalyx in animals treated with the conditioned medium was detached and missing.

These results indicate that the conditioned medium containing pro-metastatic factors can induce vascular endothelial glycocalyx degradation, suggesting that the formation of the pre-metastatic niche is accompanied by shedding of the vascular endothelial glycocalyx.

### *3.3* Cancer cells secreting pro-metastatic factors cannot directly degrade vascular endothelial glycocalyx on HUVECs in vitro

**Fig. 4:**
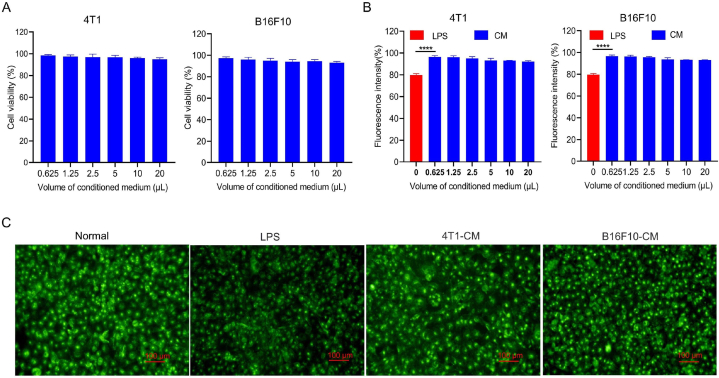
Effects of cancer cell-secreted pro-metastatic factors on HUVECs *in vitro*. A. Effect of conditioned media from 4T1 or B16F10 cell cultures on HUVEC viability in vitro. B. Effects of conditioned medium (CM) from 4T1 or B16F10 cells on the amount of vascular endothelial glycocalyx on human umbilical vein endothelial cells in culture based on fluorescent wheat germ agglutinin-labeled vascular endothelial glycocalyx. The fluorescence intensity was measured using a multifunctional enzyme labeling instrument. **C.** Representative fluorescence micrographs of cultures treated with the indicated media. Positive control cultures were based on treatment with lipopolysaccharide (LPS) to induce glycocalyx degradation. Scale bar, 20 μm ****p < 0.0001.

HUVECs are commonly used to study the function and structure of the endothelial system [52]. Therefore, these cells were selected as an appropriate cell model to study the influence of secreted pro-metastatic factors on the vascular endothelial glycocalyx. First, the safety of the conditioned medium in HUVECs was evaluated. Exposure of HUVECs to increasing amounts of conditioned medium did not reduce the cell viability to below 90% (Fig. 4A), suggesting that the medium was not cytotoxic to vascular endothelial cells. The vascular endothelial glycocalyx content in HUVECs was assayed after treatment with increasing amounts of conditioned medium. The vascular endothelial glycocalyx was labeled with FITC-labeled wheat germ agglutinin and assayed using a microplate reader. As shown in Fig. 4B, the fluorescence intensity of the positive control group (LPS) was reduced to 80% of that of the PBS group, indicating that LPS induced 20% vascular endothelial glycocalyx degradation. The fluorescence intensity of HUVECs exposed to the conditioned medium was comparable to that of the PBS group, indicating that the vascular endothelial glycocalyx was not degraded. The observation of vascular endothelial glycocalyx by fluorescence micrographs also showed that the conditioned medium group had a comparable fluorescence intensity with the normal group (Fig. 4C). This result suggests that soluble factors in the conditioned medium do not directly induce vascular endothelial glycocalyx degradation but rather alter other target cells, which in turn drive vascular endothelial glycocalyx degradation.

### 3.4 Conditioned medium from cancer cell cultures upregulates expression of heparanase in healthy mice

**Fig. 5:**
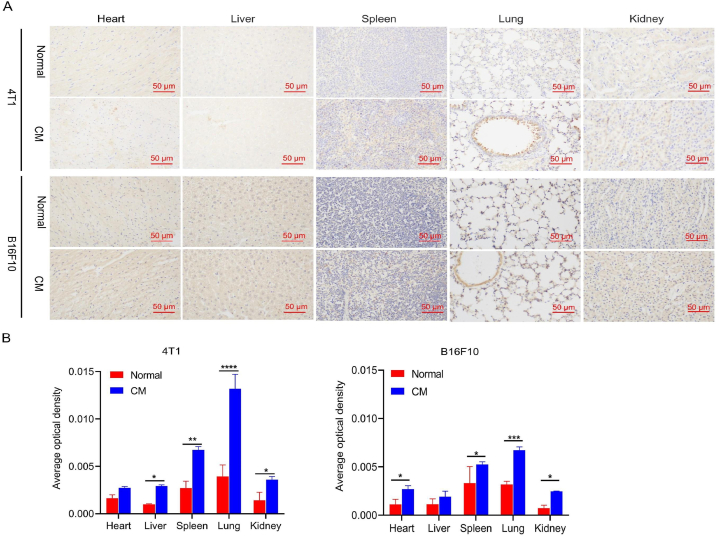
Immunohistochemical detection of heparanase in the major organs of healthy mice after injection of conditioned medium from 4T1 or B16F10 cell cultures. A. Heparinase staining of the major organs. Scale bar, 50 μm. B. Average optical density scores for heparinase expression in each organ. *p < 0.05, **p < 0.01, ***p < 0.001, ****p < 0.0001.

We further explored the mechanism of vascular endothelial glycocalyx destruction induced by the conditioned medium containing secreted pro-metastatic factors. To do so, we studied the level of heparanase, which is a heparan sulfate-specific glucuronidase directly related to vascular endothelial glycocalyx degradation [53,54]. Heparanase can shear heparan sulfate from the vascular endothelial glycocalyx, leading to its degradation and loss of function as a selective vascular permeability barrier and microcirculation regulator. Immunohistochemical analysis showed that the organs of the conditioned medium group injected with the conditioned medium exhibited higher expression of heparanase than the normal group (Fig. 5). In the lungs, the average optical density of the conditioned medium group in the two groups of mice treated with the conditioned medium was much higher than that in the normal group. This experiment confirmed that conditioned medium could induce the overexpression of heparanase, which is a specific enzyme that plays an important role in vascular endothelial glycocalyx degradation.

### 3.5 Conditioned medium from cancer cell cultures upregulates TNF-α expression in healthy mice

**Fig. 6:**
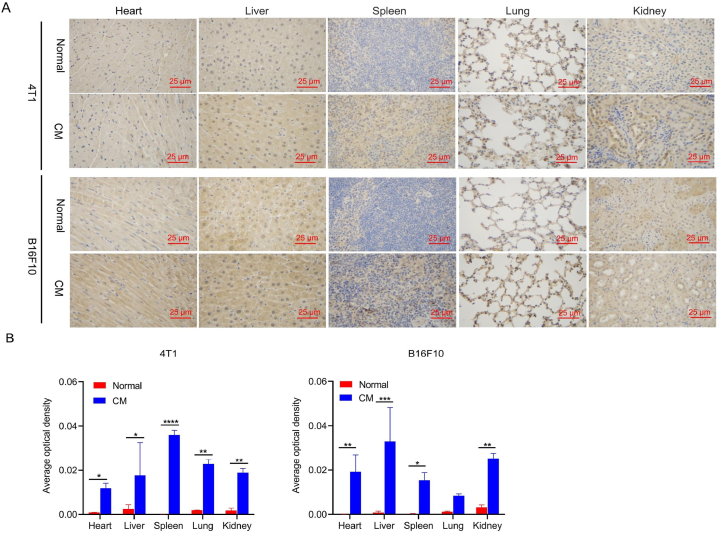
Immunohistochemical detection of TNF-α in the major organs of healthy mice after injection of conditioned medium from cultures of 4T1 or B16F10 cells. A. TNF-α staining of the major organs. Scale bar, 25 μm. B. Average optical density score of TNF-α expression in each organ. *p < 0.05, **p < 0.01, ****p < 0.0001.

Microvascular vascular endothelial glycocalyx could be rapidly degraded via TNF-α-dependent mechanisms [21]. Vascular endothelial glycocalyx degradation involved the specific loss of heparin sulfate and coincided with the activation of endothelial heparanase, which is a TNF-α-responsive, heparin sulfate-specific glucuronidase [55,56]. Here we determined the influence of conditioned medium on the expression of TNF-α in various organs. We found that after injecting conditioned medium in healthy mice, TNF-α was upregulated in all of the major organs we assayed (Fig. 6). Immunohistochemical analysis showed that TNF-α was overexpressed in all of the organs of the conditioned medium group of BALB/c and C57 mice compared to the normal group. Based on the average optical density, the expression level of TNF-α in all of the organs of the conditioned medium group was significantly higher than that of the normal group. The high level of TNF-α promotes the activation of heparanase to degrade vascular endothelial glycocalyx.

### 3.6 Macrophages play an important role in VEG degradation induced by the conditioned medium

**Fig. 7:**
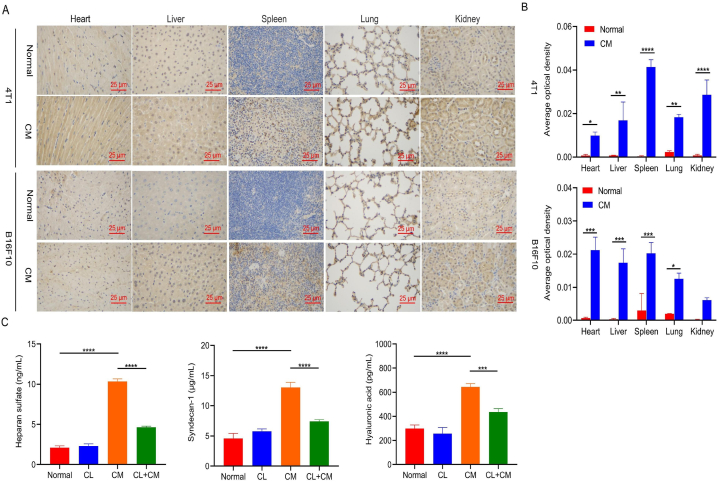
Involvement of macrophages in conditioned medium-induced degradation of vascular endothelial glycocalyx A. CD11b staining of the major organs. Scale bar, 25 μm. B. The average optical density score for CD11b expression in each organ. *p < 0.05, **p < 0.01, ***p < 0.001, ****p < 0.0001. C. Degradation of the vascular endothelial glycocalyx in mice induced by conditioned medium from 4T1 cell cultures after macrophage clearance.

The inflammatory cytokine TNF-α promotes cancer cell proliferation, migration, and invasion and is mainly secreted by macrophages [57,58]. Macrophages are major components of the tumor microenvironment that regulate various aspects of immunity. Macrophages promote invasion and metastasis from primary tumor sites by allowing cancer cells to engage in an autocrine loop that promotes cancer cell migration. Here, we evaluated the levels of the macrophage marker CD11b in various organs after injection of conditioned medium [59,60]. After injecting the conditioned medium into healthy mice, macrophage markers were upregulated in all of the major organs (Fig. 7A and B). Immunohistochemical analysis showed that CD11b was overexpressed in all organs of the conditioned medium group of BALB/c and C57 mice compared to the normal group. According to the mean optical density, the expression of CD11b in all of the organs of the CM group was significantly higher than that in the normal group. These results suggest that conditioned medium treatment enhances macrophage levels in various organs.

Through the above experiments, we found that the conditioned medium can induce the overexpression of TNF-α and CD11b, which are directly related to macrophages. We speculate that macrophages play an important role in the degradation of the vascular endothelial glycocalyx induced by the conditioned medium. To confirm this hypothesis, we depleted macrophages using a macrophage depletion kit and then assessed the effect of the conditioned medium on the degradation of the vascular endothelial glycocalyx. We found that compared to the direct injection of conditioned medium (CM), the group treated with the macrophage depletion kit (CL + CM) exhibited significantly lower levels of heparan sulfate, syndecan-1, and hyaluronic acid in the plasma (Fig. 7C). Compared with the normal group, the CL + CM group exhibited only a slight increase in the levels of vascular endothelial glycocalyx markers. This suggests that macrophages play an important role in vascular endothelial glycocalyx degradation induced by tumor-conditioned medium.

## 4 Discussion and conclusion

According to the literature, the injection of CM containing extracellular vesicles and soluble factors into mice results in the formation of pre-metastatic niches in mice [41]. Analysis of the molecular mechanism of pre-metastatic niche formation has shown that soluble factors secreted by orthotopic tumors are key factors in the recruitment of bone marrow-derived suppressor cells during metastasis. In addition, extracellular vesicles from tumors play an important role in promoting tumor growth and metastasis because they contain important biological information regarding tumor cells. Extracellular vesicles and soluble factors produced by orthotopic tumor cells are co-induced and can directly participate in the remodeling of the local vascular bed, recruitment of bone marrow-derived suppressor cells, and rearrangement of the matrix to form a pre-metastatic niche [61].

Using two mouse models, we provide evidence linking the formation of pre-metastatic niches to vascular endothelial glycocalyx degradation, which may help explain how these niches facilitate the intra- and extravasation of circulating tumor cells into and out of the circulation, thereby promoting tumor invasion into secondary tissues. We induced the formation of pre-metastatic niches and vascular endothelial glycocalyx degradation using only sterile-filtered conditioned medium from cultured cancer cells, implying that primary cancer cells induce these effects by secreting soluble factors. As we were unable to replicate these effects in HUVEC cultures, it appears that the soluble factors did not induce significant vascular endothelial glycocalyx degradation but rather upregulated or downregulated genes in target cells to produce enzymes and/or other proteins that mediate these effects. Indeed, we found that the conditioned medium upregulated expression of the vascular endothelial glycocalyx-degrading enzyme heparanase and the closely related TNF-α and induced inflammation in the major organs of healthy mice. We found that plasma levels of vascular endothelial glycocalyx markers in mice injected with the conditioned medium after macrophage clearance were significantly lower than those in mice injected directly with the conditioned medium. We speculate that the conditioned medium does not directly cause vascular endothelial glycocalyx degradation but induces heparanase overexpression by promoting macrophages to secrete a large amount of TNF-α, and this enzyme completes the degradation of vascular endothelial glycocalyx.

According to the results, all organs had obvious vascular endothelial glycocalyx shedding. In particular, the lungs exhibited severe vascular endothelial glycocalyx shedding. This is consistent with the fact that many types of cancer metastasize to the lungs more than to other tissues [36,62]. Tumor metastasis is not only related to glycocalyx shedding but also to extracellular vesicles and soluble factors produced by orthotopic tumors. These extracellular vesicles and soluble factors carry important biological information about tumor cells and are directly involved in the remodeling of the local vascular bed, enrolment of immune cells, and rearrangement of the matrix, which promote lung and liver metastasis of tumor cells [63].

In addition, we investigated whether conditioned medium from different cancer cell lines induced vascular endothelial glycocalyx shedding. The injection of B16F10 or 4T1 conditioned media induced obvious inflammation in various organs (Fig. 2). In addition, the levels of all three vascular endothelial glycocalyx degradation components (heparan sulfate, hyaluronic acid, and syndecan-1) in the two mouse blood samples were compared (Fig. 3). These results indicate that B16F10 and 4T1 conditioned medium can induce the same degree of vascular endothelial glycocalyx degradation.

Our experiments lead us to suggest a sequence of events whereby soluble factors from primary tumors stimulate local inflammatory responses in secondary tissues, as well as infiltration of macrophages which secrete large amounts of TNF-α, and these responses upregulate heparanase, leading to vascular endothelial glycocalyx degradation. This permeabilizes blood vessels and promotes the intra- and extravasation of circulating tumor cells. Therefore, this pathway should be explored in a range of cancer types and preclinical models. Such research may strengthen the case that, in addition to chemotherapy and surgery to attack tumors directly [64,65], anticancer therapies should target the formation of pre-metastatic niches [66,67]. Given the importance of the endovascular calyx in tumor metastasis, it may be possible to repair the damaged endovascular calyx to inhibit the pre-metastatic niche and tumor metastasis. Various methods have been reported for repairing the vascular endothelial glycocalyx. One method is inhibition of the activity of the enzymes that cause degradation of the vascular endothelial glycocalyx, such as by using drugs such as antithrombin and dexmedetomidine to inhibit the activity of heparinase and hyaluronidase [68,69], and inhibition of vascular endothelial glycocalyx degradation. Another is supplementation of the main components of the glycocalyx, such as N-acetylglucosamine [70], which can promote the biosynthesis of the vascular endothelial glycocalyx and thus repair vascular endothelial glycocalyx damage [71]. This may provide a new approach to blocking tumor metastasis.

Above all, our findings suggest that the formation of pre-metastatic niches involves degradation of the vascular endothelial glycocalyx, which may hence be a useful target for developing therapies to inhibit cancer metastasis.

### Ethics statement

All animal experiments were performed in accordance with the principles of care and use of laboratory animals and approved by the Experimental Animal ethics Committee of Chengdu University (82073803).

## CRediT authorship contribution statement

**Rui Qu:** Writing – original draft, Project administration, Methodology, Investigation, Data curation. **Wenxuan Du:** Writing – original draft, Project administration. **Shuyao Li:** Writing – original draft, Project administration. **Wei Li:** Writing – review & editing, Methodology, Investigation, Conceptualization. **Guangfei Wei:** Writing – review & editing, Funding acquisition. **Zhoujiang Chen:** Writing – review & editing, Data curation. **Huile Gao:** Writing – review & editing, Conceptualization. **Sanjun Shi:** Writing – review & editing, Methodology. **Liang Zou:** Writing – review & editing, Supervision, Conceptualization. **Hanmei Li:** Writing – review & editing, Funding acquisition, Conceptualization.

## Declaration of competing interest

The authors declare that they have no conflict of interest.
